# A challenge in diagnosis and management of ulcerative colitis in elderly patient with atypical presentation: A reported case

**DOI:** 10.1016/j.ijscr.2019.07.008

**Published:** 2019-07-16

**Authors:** Panutchaya Kongon, Vorapatu Tangsirapat, Vittawat Ohmpornuwat, Kannakrit Sumtong, Vichack Chakrapan Na Ayudhya, Kobkool Chakrapan Na Ayudhya, Paiboon Sookpotarom, Paisarn Vejchapipat

**Affiliations:** a Department of Surgery, Panyananthaphikkhu Chonprathan Medical Center, Srinakharinwirot University, Nonthaburi, 11120, Thailand; b Department of Surgery, Faculty of Medicine, Chulalongkorn University, Bangkok, 10330, Thailand

**Keywords:** Ulcerative colitis, Peritonitis, Elderly patient

## Abstract

**Introduction:**

Recognition of elderly-onset ulcerative colitis (UC) remains poor as the differential diagnosis in older patients with acute abdominal pain and bloody diarrhea is extensive and UC is generally not the obvious cause. A typical presentation in an elderly patient with acute severe UC can mimic surgical abdomen.

**Presentation of case:**

An 80-year-old female had been presented with high grade fever, abdominal pain and diarrhoea. Physical examination showed sign of peritonitis and severe hypotension. A provisional diagnosis of perforated sigmoid diverticulitis was made. However, at the theater, there was only turbid yellowish ascites at cal-de-sac. Consequently, colonoscopy was performed and revealed continuous and circumferential erythematous with friable mucosa and multiple shallow ulcer along upper left side colon. Histologic examination of the colonic tissue was consistent with UC.

**Discussion:**

As UC is uncommon in Thailand and clinical features of elderly-onset UC are much more non-specific; as a result, misdiagnosis at initial presentation is more common in elderly patients (60%) than that in younger population (15%). These might result in an unnecessary exploratory laparotomy; however, computed tomography scan can reduce the risk of that event.

**Conclusion:**

The diagnosis of severe acute UC in elderly patients with acute abdomen had been complicated by the distinctive physiology of this aged group with atypical presentation and markedly unreliable physical examination. Eventually, severe UC should always be kept in mind with a circumstance of abdominal pain in geriatric population.

## Highlights

• Diagnosis of severe UC constitutes a difficult task in elderly patient.
• CT imaging can help physicians exclude non-surgical condition.
• Severe UC should be kept in mind in elderly patients with new onset abdominal pain.

## 1 Introduction

Ulcerative colitis (UC), collectively known as inflammatory bowel disease, is a common cause of chronic gastrointestinal disease involving mucosal surface of large intestine. UC has a bimodal age distribution with 10–30% of the affected population older than 60 years [1]. However, recognition of elderly-onset UC remains poor as the differential diagnosis in older patients with acute abdominal pain and bloody diarrhea is extensive and UC is generally not the obvious cause. As UC is uncommon in Thailand and only few reports have been published in the literature, confirmation of the diagnosis at initial presentation, particularly in an elderly patient, is somewhat more difficult which usually results in a delay or misdiagnosis [2]. A typical presentation in an elderly patient with acute severe UC mimicking surgical abdomen in this reported case led to an unnecessary exploratory laparotomy instead of treatment with parenteral corticosteroid.

This work is compliant with the SCARE checklist, and also, has been reported in line with the SCARE criteria [3].

## 2 Presentation of case

An 80-year-old female patient came to our hospital after suffering from diarrhoea, loss of appetite, fatigue and weakness for three months. She also had a high-grade fever for 3 days. Physical examination confirmed the high-grade fever and sign of severe dehydration. A per rectum examination revealed yellow stool. There was a presence of abdominal distention and generalized tenderness with marked tenderness at left side. Laboratory tests revealed a total white blood cell count of 11,000 per microliter and a neutrophil count of 80% also with high rising of creatinine and low glomerular filtration rate. Abdominal radiography showed mild dilatation of left-sided colon with no pneumoperitoneum. The initial clinical diagnosis of left side colitis was made. She was admitted for intravenous antibiotic and scheduled to investigate with computerized tomography (CT) scan of whole abdomen after adequate resuscitation with aggressive intravenous hydration.

**Fig. 1:**
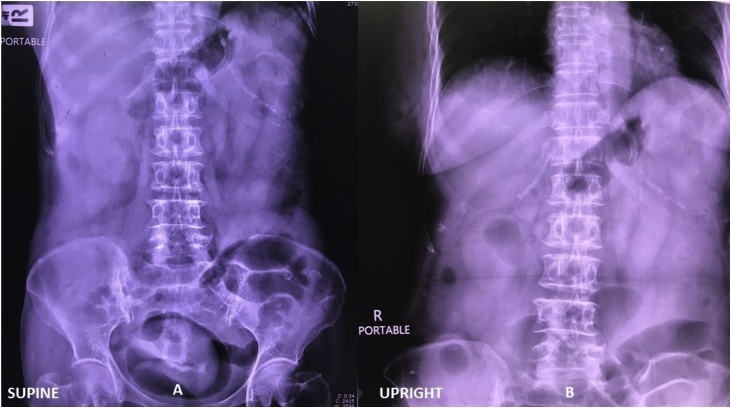
Plain abdominal radiography showed dilated small at left side abdomen in supine (A) and upright (B) view.

**Fig. 2:**
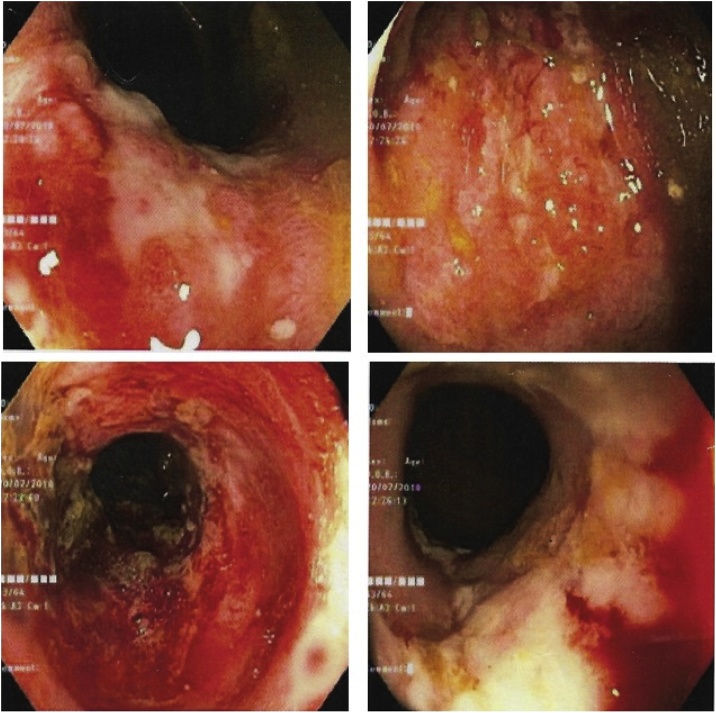
Endoscopic findings as continuous and circumferential erythematous with friable mucosa and multiple shallow ulcer along upper left side colon was consistent with UC.

Three hours following the admission; however, the patient developed severe hypotension and abdominal pain. The patient’s vital signs were as follows: blood pressure of 80/50 mmHg, heart rate of 120/min and temperature of 39 °C. Her abdominal signs had gotten worse revealing increased distension, generalized involuntary guarding and marked tenderness at the left side of the abdomen. Abdominal radiography showed dilated small bowel at left side abdomen (Fig. 1). Even after a large volume of isotonic saline solution and vasopressors, her blood pressure was not improved. Since her condition was unstable and accompanied by low level of creatinine clearance, CT scan was not performed. A provisional diagnosis of perforated sigmoid diverticulitis was made. Consequently, the patient was transferred to operating room. At the theatre, there was a presence of turbid yellowish ascites 100 mL at cal-de-sac region. There was no dilatation of sigmoid colon and also no intestinal perforation. Abdominal toilet was performed to clear the yellowish ascites at cul-de-sac. Post-operatively, the clinical including fever and abdominal tenderness had improved. The patient was eventually discharged on post-operative day 10. However, following 1 week of follow-up, on out-patient examination, she still had a frequency of stool. The stool examination at this time showed positive for red blood cells, thereby being appropriate for colonoscopy. The investigation revealed continuous and circumferential erythematous with friable mucosa with multiple shallow ulcer along upper left side colon (Fig. 2). A piece of tissue retrieved for biopsy was granular and friable. Histologic examination of the colonic tissue was suspected of ulcerative colitis. Cytomegalovirus, acid-fast bacilli and Gomori Methenamine-Silver Nitrate Stains were all negative. Following a discussion for any risks counterweighted with potential benefits of starting anti-inflammatory drug, patient and her relatives refused to have anti-inflammatory drugs because of poor performance status and her comorbidities. As a result, the only treatment was oral antibiotics (ciprofloxacin and metronidazole) and closed monitoring by frequent follow-up for early detection and prevention of any disease complications.

## 3 Discussion

Clinical presentation in UC can vary from mild to severe. In a small number of patients with acute severe form (15%), they suffer a fulminant course in which the presentation much likes an acute surgical abdomen and the condition is life-threatening which can be associated with significant morbidity [[4], [5], [6]]. Generally, elderly patients with UC appear to have less complicated and milder disease course compared with younger patients at initial presentation [7,8]. In this population, acute severe UC was very rare; however, 74% of all severe cases required surgery and all of them had surgical causes with a mortality rate close to 25% at 3 months [6]. As previously mentioned, diagnosis of acute severe UC in our patient faced a significant and challenging problem, not only did she had atypical symptoms and signs that mimicked a condition requiring surgery, but the surgical finding also did not reveal any surgical causes.

The clinical features of elderly-onset UC are much more non-specific; as a result, misdiagnosis at initial presentation is more common in elderly patients (60%) than that in younger population (15%) [9]. These may be explained by other more prevalent disorders that should initially be considered in elderly patients presenting with new abdominal symptoms. The differential diagnosis should include colorectal malignancy and colonic diverticulitis. Colonoscopy and mucosal biopsy are the principal methods for the diagnosis of UC [10]. Unfortunately, the patient did not have microscopic or macroscopic blood seen in the stool and the initial abdominal signs was likely to be peritonitis, she was then treated with surgery in lieu of medicine. But fortunately, following the surgery, nothing would happen and finally there was a presence of positive signs through her feces. That had brought her to an appropriate investigation. Like our patient, the typical endoscopic findings in patients with UC include edematous mucosa, erythema, loss of vascular marking, mucosal friability, erosion and ulceration [11]. Although CT scan is not recommended as a primary means of diagnosis of UC because of its low diagnostic sensitivity for early disease and often the results are normal [12], it is useful imaging technique for exclusion of conditions that require immediate surgical intervention. As in our patient in which the provisional diagnosis was suitable for CT scan; however, according to our patient conditions, CT imaging could not be performed.

With respect to the Truelove and Witt’s criteria that acute severe UC with a life threatening condition characterized by presence of more than 6 bloody stools/day along with any one of the following: tachycardia >90 beat per min, fever >37.8 °C, hemoglobin <10.5 g/dL, and/or erythrocyte sedimentation rate >30 mm/h [[13], [14], [15]], the prompted treatment with parenteral corticosteroid was remains the first-line therapy. Unfortunately, our patient conditions were not improved with hydration resuscitation and vasopressor drugs, the then therapy was to perform surgery rather than this specific treatment.

The role of antibiotics in the primary treatment of UC is controversial, some study proposed the role of antibiotics as a therapy for UC to induce remission in an active disease and to prevent relapse. They are usually associated with a modest improvement [16]. The plausible explanation for the improvement of our patient might be explained by this factor. However, long term follow-up to determine whether antibiotic actually has a role to induce remission in this disorder is needed for this group of patients.

## 4 Conclusion

The diagnosis of severe acute UC in elderly patients with acute abdomen is still challenging. Not only is it uncommon in Asia compared to Western, but the distinctive physiology of this aged group with atypical presentation and markedly unreliable physical examination also lead to a difficulty in diagnosis. CT imaging is a diagnostic tool which can help physicians exclude non-surgical condition and reduce rate of negative surgery. Eventually, severe UC should always be kept in mind with a circumstance of abdominal pain in geriatric population.

## Declaration of Competing Interest

The authors declare that there is no conflict of interest regarding the publication of this article.

## Sources of funding

This work received no funding.

## Ethical approval

The consent form and information sheet using in the process of obtaining a consent were approved by IRB at our institution.

## Consent

The patient has been informed prior to the conduction of this manuscript and informed consent has also been obtained. A copy of the written consent is available for review by the editor-in-chief of the journal on request.

## Author’s contribution

Panutchaya Kongon and Vorapatu Tangsirapat collected data and wrote manuscript.

Vittawat Ohmpornuwat, Kannakrit Sumtong, Vichack Chakrapan Na Ayudhya and Kobkool Chakrapan Na Ayudhya contributed to conceptualization.

Paiboon Sookpotarom contributed to conceptualization, data curation, supervision and editing of the manuscript.

Paisarn Vejchapipat finally edited this manuscript.

## Registration of research studies

It seems not necessary for this presentation since this is not a first-in-man case report.

## Guarantor

Panutchaya Kongon.

## Provenance and peer review

Not commissioned, internally peer-reviewed.
